# Clinical Effects of Exercise Rehabilitation Combined with Repaglinide in the Treatment of Diabetes

**DOI:** 10.1155/2022/6309188

**Published:** 2022-03-25

**Authors:** Yan Li, Xi Wang, Ying Zhang

**Affiliations:** ^1^Department of Nursing, China-Japan Union Hospital of Jilin University, Changchun, China; ^2^Department of Gynecology, China-Japan Union Hospital of Jilin University, Changchun, China; ^3^Department of Cardiology, China-Japan Union Hospital of Jilin University, Changchun, China

## Abstract

**Objective:**

Diabetes, a common endocrine and metabolic disease in clinical practice, generally manifests a certain defect in insulin secretion due to several factors, thereafter leading to a metabolic disorder such as hyperglycemia. This study was conducted to explore the clinical effects of repaglinide combined with exercise rehabilitation on improving the blood glucose of patients with diabetes.

**Methods:**

In this retrospective study, 100 patients with diabetes treated in our hospital from January 2018 to January 2020 were assessed for eligibility and recruited. They were assigned at a ratio of 1 : 1 to receive either repaglinide (control group) or repaglinide plus exercise rehabilitation (experimental group). Outcome measures include fasting blood glucose, 2 h postprandial blood glucose, glycosylated hemoglobin, time to normal blood glucose, blood glucose fluctuation, insulin dosage, adverse reactions, and blood glucose adequate rate.

**Results:**

All eligible patients showed similar pretreatment fasting blood glucose, glycosylated hemoglobin, and 2 h postprandial blood glucose (*P* > 0.05). After treatment, repaglinide plus exercise rehabilitation resulted in lower levels of fasting blood glucose, glycosylated hemoglobin, and 2 h postprandial blood glucose versus repaglinide alone (*P* < 0.05). Repaglinide plus exercise rehabilitation was associated with a significantly shorter time to normal blood glucose and a milder fluctuation versus repaglinide (*P* < 0.05). The incidence of adverse reactions and blood glucose adequate rate was 6% and 94% in the experimental group and 50% and 52% in the control group, respectively (*P* < 0.05).

**Conclusion:**

Repaglinide plus exercise rehabilitation results in effective blood glucose control and reduced incidence of adverse reactions and yields a promising efficacy, so it is worthy of clinical promotion and application.

## 1. Introduction

Diabetes, a common endocrine and metabolic disease in clinical practice, generally manifests a certain defect in insulin secretion due to several factors, thereafter leading to a metabolic disorder such as hyperglycemia [1]. The elderly population is susceptible to diabetes mellitus due to reduced body functions [2]. The high blood glucose elicited by diabetes is considered detrimental to the patients' multiple organs, nervous system, and vascular system, resulting in the complications such as diabetic retinopathy, diabetic nephropathy, and diabetic foot [3–5]. At present, most clinical treatments are carried out by oral medication and insulin subcutaneous injection, coupled with self-management measures such as diet adjustment, exercise, and blood glucose monitoring. With the feature of rapid action, repaglinide lowers the blood glucose by inhibiting insulin release from the pancreas [6–8]. It is associated with short-term but promising effects on postprandial blood glucose but fails to yield a satisfactory control on fasting blood glucose. Exercise rehabilitation improves the sensitivity of insulin target cells to insulin and glucose utilization and thus contributes to lower blood glucose [9–11]. This study was aimed at exploring the clinical effects of repaglinide combined with exercise rehabilitation on improving the blood glucose of patients with diabetes.

## 2. Materials and Methods

### 2.1. General Materials

In this retrospective study, 100 patients with diabetes treated in our hospital from January 2018 to January 2020 were assessed for eligibility and recruited. They were assigned at a ratio of 1 : 1 to a control group (*n* = 50) or experimental group (*n* = 50). The baseline characteristics of the control group (27 males and 23 females, course of disease of 2-6 years, mean course of disease of 3.6 ± 0.5, aged 35–73 years, and mean age of 50.62 ± 3.4 years) were comparable with those of the experimental group (29 males and 21 females, course of disease of 2.5-7 years, mean course of disease of 3.3 ± 0.4, aged 36–75 years, and mean age of 51.6 ± 3.2 years) (*P* > 0.05).

### 2.2. Inclusion Criteria

The following are the inclusion criteria: (1) patients who met the diagnostic criteria of diabetes; (2) patients without a history of severe hypoglycemia; (3) patients who stopped using other types of hypoglycemic drugs at one month before enrollment; (4) patients with certain cognitive and communication skills; and (5) the ethics committee of our hospital had approved this study, and patients and their families were informed of the purpose and process of this research and provided written informed consent.

### 2.3. Exclusion Criteria

The following are the exclusion criteria: (1) patients with serious insufficiency of other combined organs such as the heart, kidney, and liver; (2) patients with malignant tumors; (3) patients with limb movement disorders; (4) patients with poor compliance; and (5) patients with drug allergies to the drugs used in this study.

### 2.4. Methods

The control group was given repaglinide (Beijing Wansheng Pharmaceutical Co., Ltd., SFDA approval number H20133037) orally at 15 minutes before meals, 0.5 mg/time, 3 times/d. The dosage was modified based on 2 h postprandial plasma glucose (2hPG) within a maximum dosage of 2 mg. The eligible patients in the experimental group were treated with repaglinide plus exercise rehabilitation after discharge. The treatment was performed in wake of the evaluation of the patients' exercise function and cardiopulmonary function. A week of adaptive training was carried out before the formal exercise. The exercise was performed and progressively strengthened and reasonably modified based on their conditions. Before the formal intervention one week later, the patients were instructed to perform 5-10 minutes of low-intensity barehanded exercises or slow walking. Formal training mainly consists of fast and slow walking. The patients were equipped with a heart rate monitor throughout the whole exercise that lasted 30 minutes, and the heart rate was controlled at 50%-70% of the highest heart rate (220 ages), followed by 5-10 minutes of restorative exercise-slow walking or barehanded exercises. Patients started exercise about one hour after a meal and wore a name card with their name, disease, and contact on it for emergency needs. Candies or biscuits were allowed to prevent palpitation, fatigue, and hunger. Exercises were performed with an interval of less than one day and each exercise lasting 40-60 min, four times per week. The exercise rehabilitation training of the patients was closely monitored, and the patients' conditions were evaluated once a week. An exercise diary of each patient was a must for data management and analysis.

All the measurements implemented in the two groups continued for 6 months.

### 2.5. Evaluation Criteria

On the evening before each study day, patients consumed a meal before 7 pm. After the meal, patients fasted from solids and liquids (14 h for solids and 12 h for liquids) until the following morning. On the study day, patients attended the department at 8 am, and an intravenous catheter was inserted for blood sampling.

In this study, the fasting plasma glucose (FPG), 2hPG, glycosylated hemoglobin (HbA1c), and the blood glucose adequate rate of the two groups before and after treatment were analyzed (normal levels of FPG and 2hPG were <5.1 mmol/L and <8.5 mmol/L, respectively).

### 2.6. Statistical Methods

In this study, SPSS20.0 was used for data analyses, and GraphPad Prism 7 (GraphPad Software, San Diego, USA) was for image rendering. The enumeration data were represented by *n* (%) and processed using the *X*^2^ test. The measurement data were represented by $\bar{x}\pm s$ and processed by the *t*-test. *P* < 0.05 indicated that the difference was statistically significant.

## 3. Results

### 3.1. Comparison of FPG

**Figure 1:**
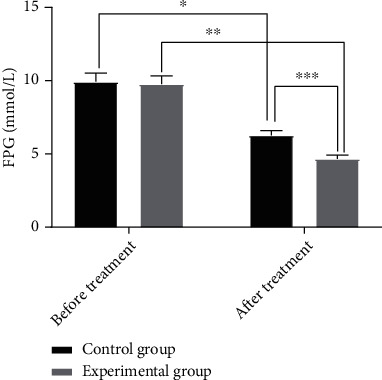
Comparison of FPG before and after treatment (*x* ± *s*). The *x*-axis indicates before and after treatment, and the *y*-axis indicates the FPG level (mmol/L). The FPG levels of patients in the control group before and after treatment were 9.52 ± 0.83 mmol/L and 6.03 ± 0.47 mmol/L, respectively. The FPG levels of patients in the experimental group before and after treatment were 9.38 ± 0.78 mmol/L and 4.49 ± 0.36 mmol/L, respectively. ∗ indicates that there is a significant difference in FPG levels before and after treatment in the control group (*t* = 25.8724, *P* = 000). ∗∗ indicates that a significant difference is found in FPG levels before and after treatment in the experimental group (*t* = 40.2500, *P* = 000). ∗∗∗ indicates that a significant difference is observed in FPG levels between the experimental group and the control group after treatment (*t* = 18.3934, *P* = 000).

There was no statistical significance in the FPG difference between the two groups before treatment (*P* > 0.05). After treatment, repaglinide plus exercise rehabilitation resulted in lower FPG levels versus repaglinide alone (*P* < 0.05). See Figure 1.

### 3.2. Comparison of 2hPG

**Figure 2:**
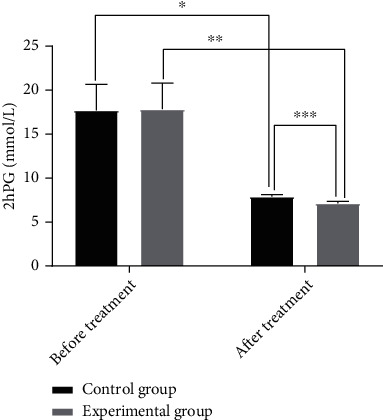
Comparison of 2hPG before and after treatment (*x* ± *s*). The *x*-axis indicates before and after treatment, and the *y*-axis indicates the 2hPG (mmol/L). The 2hPG levels of patients in the control group before and after treatment were 15.64 ± 4.16 mmol/L and 7.78 ± 0.27 mmol/L, respectively. The 2hPG levels of patients in the experimental group before and after treatment were 15.73 ± 4.22 mmol/L and 6.97 ± 0.33 mmol/L, respectively. ∗ indicates that a significant difference is found in 2hPG levels before and after treatment in the control group (*t* = 13.3322, *P* = 000). ∗∗ indicates that a significant difference is observed in 2hPG levels before and after treatment in the experimental group (*t* = 14.6337, *P* = 000); ∗∗∗ indicates that a significant difference is discovered in 2hPG levels between the experimental group and the control group after treatment (*t* = 13.4330, *P* = 000).

The difference in 2hPG between the two groups before treatment was not statistically significant (*P* > 0.05), and the two groups both decreased in 2hPG after treatment, and the experimental group had significantly lower 2hPG as compared to the control group (*P* < 0.05). See Figure 2.

### 3.3. Comparison of HbA1c

**Figure 3:**
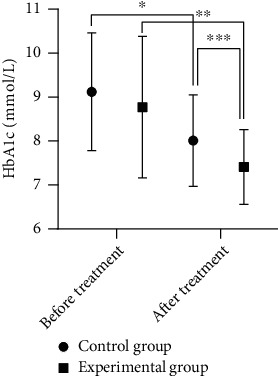
Comparison of HbA1c between the two groups of patients before and after treatment. The abscissa indicates before and after treatment, and the ordinate indicates the level of HbA1c, in mmol/L. The HbA1c levels of patients in the control group before and after treatment were 9.12 ± 1.34 mmol/L and 8.01 ± 1.04 mmol/L, respectively. The HbA1c levels of patients in the experimental group before and after treatment were 8.77 ± 1.61 mmol/L and 7.41 ± 0.85 mmol/L, respectively. ∗ indicates that there is a significant difference in HbA1c levels before and after treatment in the control group (*t* = 4.6272, *P* = 000). ∗∗ indicates significant differences in HbA1c levels before and after treatment in the experimental group (*t* = 5.2821, *P* = 000). ∗∗∗ indicates that there is a significant difference in HbA1c levels between the experimental group and the control group after treatment (*t* = 3.1587, *P* = 000).

The HbA1c difference between the two groups of patients before treatment was not statistically significant (*P* > 0.05). The experimental group showed significantly decreased levels of HbA1c versus the control group (*P* < 0.05). See Figure 3 for details.

### 3.4. Comparison of the Time to Normal Blood Glucose, Blood Glucose Fluctuation, and Insulin Dosage

After treatment, repaglinide plus exercise rehabilitation was associated with a significantly shorter time to normal blood glucose and a milder fluctuation versus repaglinide (*P* < 0.05). See Table 1.

### 3.5. Comparison of Blood Glucose Adequate Rate

After treatment, the blood glucose adequate rate and nonadequate rate were 26 (52%) and 24 (48%) of patients in the control group and 47 (94%) and 3 (6%) of those in the experimental group. There is a significant difference in the adequate rate between the two groups after treatment (*X*^2^ = 22.3744, *P* ≤ 0.001).

### 3.6. Comparison of Adverse Reactions

The incidence of adverse reactions was 6% in the experimental group and 50% in the control group, respectively (*P* < 0.05). See Table 2.

## 4. Discussion

Diabetes is induced by genetic and environmental factors, with the manifestation of high blood glucose [12, 13]. As the disease aggravates, the incidence of complications such as microvascular disease, neuropathy, and atherosclerosis also rises. Diabetes has now become the third high-risk disease that threatens the life and health of patients after cardiovascular diseases and cancers. Early detection and treatment are of great significance to the treatment and prognosis of patients [14, 15].

Repaglinide, an insulin secretion agent, improves the pancreatic *β*-cell function by exerting the dependence on glucose and specifically promotes insulin secretion in the early stage. High glucose results in strong effects on pancreatic *β*-cells. The duration of action of repaglinide is within 30 minutes. In the early stage of abnormal insulin secretion, repaglinide can minimize the incidence of postprandial hyperglycemia, without impact on the cardiovascular system, but reduces the risk of cardiovascular damage. The administration of repaglinide is considered safe in the elderly with comparatively weak cardiovascular systems [12, 16]; however, its efficacy on FPG control before meals is poor. Exercise rehabilitation is to accelerate the fat metabolism and reduce the psychological burden of patients through appropriate exercises, which also effectively reduces medication costs and improves their weight, thereby enhancing the overall immunity of patients. It also maintains a stable in vivo environment by increasing the number of insulin receptors in muscle cells and its sensitivity to insulin [17].

The study of Suzuki et al. [18] showed that comprehensive repaglinide therapy can effectively recover the FPG, HbA1c, and 2hPG of the patients to a normal level. In the present study, repaglinide plus exercise rehabilitation resulted in lower levels of fasting blood glucose, glycosylated hemoglobin, and 2 h postprandial blood glucose versus repaglinide alone (*P* < 0.05). Repaglinide plus exercise rehabilitation was associated with a significantly shorter time to normal blood glucose and a milder fluctuation versus repaglinide (*P* < 0.05). The incidence of adverse reactions and blood glucose adequate rate was 6% and 94% in the experimental group and 50% and 52% in the control group, respectively. Studies have shown that repaglinide plus exercise rehabilitation therapy effectively maintains the stability of FPG and postprandial glucose. During treatment, it can improve the compliance of patients and increase the blood glucose adequate rate. Furthermore, joint therapy also reduces adverse reactions. Neveen et al. [19] pointed out that aerobic exercise contributes to the control of blood glucose and blood lipids of patients, which is similar to the results of the present study.

In summary, repaglinide plus exercise rehabilitation results in effective blood glucose control and reduced incidence of adverse reactions and yields a promising efficacy, so it is worthy of clinical promotion and application.

## Figures and Tables

**Table 1:** Comparison of the compliance time and fluctuation value of blood glucose and insulin dosage after treatment (*x* ± *s*).

Group	Number	Time to reach the standard (d)	Fluctuation value (mmol/L)	Insulin dosage (U/d)
Control group	50	5.67 ± 1.05	7.54 ± 1.68	45.71 ± 7.06
Experimental group	50	4.08 ± 0.97	5.31 ± 1.04	40.06 ± 6.33
*t*	7.8651	7.9806	4.2133	
*P*		≤0.001	≤0.001	≤0.001

**Table 2:** Comparison of adverse reactions during treatment [*n* (%)].

Group	Numbers	Hypoglycemia	Hyperglycemia	Allergies	Adverse reactions
Control group	50	8 (16.00)	11 (22.00)	6 (12.00)	25 (50.00)
Experimental group	50	2 (4.00)	0 (0.00)	1 (2.00)	3 (6.00)
*X* ^2^					25.6921
*P*					<0.05

## Data Availability

The datasets used during the present study are available from the corresponding author upon reasonable request.
